# The Iron Age Dogs from Alaybeyi Höyük, Eastern Anatolia

**DOI:** 10.3390/ani11041163

**Published:** 2021-04-18

**Authors:** Abu B. Siddiq, Vedat Onar, Rıfat Mutuş, Dominik Poradowski

**Affiliations:** 1 Department of Anthropology, Mardin Artuklu University, Mardin 47200, Turkey; abubakarsiddiq@artuklu.edu.tr; 2 Osteoarchaeology Practice and Research Centre and Department of Anatomy, Istanbul University-Cerrahpaşa, Istanbul 34320, Turkey; onar@istanbul.edu.tr; 3 Faculty of Health Sciences, Istanbul Gelişim University, Istanbul 34310, Turkey; rmutus@gelisim.edu.tr; 4 Department of Biostructure and Animal Physiology, Wroclaw University of Environmental and Life Sciences, Kożuchowska 1, 51-631 Wrocław, Poland

**Keywords:** *Canis lupus familiaris*, Alaybeyi Höyük, Iron Age, human–dog relationship, Eastern Anatolia

## Abstract

**Simple Summary:**

Zooarchaeological studies on canine skeletal remains are rare. Faunal assemblages from the Near East, including Anatolia, give us a valuable source of information about the role of dogs in the Iron Age society. In the 2016 and 2017 excavations at Alaybeyi Höyük (Eastern Anatolia), over 300 dog bones were unearthed from Iron Age buildings and workshop complex. This study examined 143 specimens that were accessible for morphometric analysis. The zooarchaeological analysis proved that the majority of them came from nine individuals. The dentition and epiphyseal bone fusion further allowed their age estimation (8 adults and 1 juvenile). Two individuals were identified as males and one as female, but the sex of the other individuals was indeterminate. The height at withers estimations and their comparison with other archaeological dogs in Eastern Anatolia as well as modern dog breeds showed that Alaybeyi dogs were significantly larger and heavier. It is also worth highlighting the presence of butchering marks resulting from the consumption of dog meat. Yet, it appeared that cynophagia had only occurred occasionally at Alaybeyi Höyük.

**Abstract:**

To date, little is known about the biological and cultural status of Iron Age dogs in Anatolia. Here, we present a zooarchaeological study of an assemblage of 143 Iron Age dog bones, including two dog skeletons, unearthed from the 2016 and 2017 salvage excavations at Alaybeyi Höyük, Eastern Anatolia. At least eight adults and one juvenile individual, along with a large number of miscellaneous specimens, were identified. The morphological status of the Alaybeyi dogs were primarily compared to previously published Iron Age dogs from Yoncatepe in Eastern Anatolia, and with the average mean of 18 modern dog breeds. Unlike in other Eastern Anatolian Iron Age sites, butcher marks were observed in some specimens, indicating at least occasional cynophagy at the site. Noticeable pathologies were found in about 5% of the sample, particularly pathologies of the oral cavity and dentitions, suggesting that some of the dogs at Alaybeyi Höyük might have been undernourished, had to live on solid food, and probably injured by humans. The results of this study reflect both the morphological and biological status of Alaybeyi dogs, as well as the Alaybeyi people’s attitudes toward dogs, adding vital information to the very limited archaeological knowledge of dogs in Anatolia.

## 1. Introduction

Dogs were the first domestic animals in the world. Genetic evidence suggests that there were multiple lineages of dog domestication in different parts of Eurasia by at least 14,000–15,000 years ago [1,2]. By the Holocene, domestic dogs appeared in different parts of the world, and dog burials were discovered from house foundation deposits, special activity areas, or associated with human burials [3,4,5,6,7]. In the Near East, dogs were present prior to the Neolithic, helping the Late Pleistocene foragers in hunting and protection [7,8]. Since the beginning of sedentary life, dog bones sporadically appear at the majority of the Early Neolithic and most of the Late Neolithic settlements in Anatolia [5,9,10,11]. In many settlements, dogs were buried alone, and sometimes buried with humans and other animals [5,11,12,13,14,15,16,17]. In others, dog bones were unceremoniously discarded in general waste deposits [11,18,19,20]. Dogs were also associated with fertility deities, healing, the underworld, and the afterlife in Anatolia—evident from multiple cases of dog burials and ritual sacrificing of dogs [13,21].

Although sporadic, most of the Bronze Age and Iron Age sites in Eastern Anatolia also represented dog bones, often associated with both household buildings and necropolis areas [14,17,20,22,23]. In the western part of Eastern Anatolia, the Bronze Age layers of Arslantepe presented an unusual increase in dogs, comprised of up to 10.5% of the total identified fauna [22]. However, the following Iron Age layers at the site revealed very sporadic dog bones [12]. A series of sites in the Elazığ region also represented a large number of dog bones, often comprised of up to 2% of the total identified fauna, from the Bronze Age levels, but very fewer dog bones from the Iron Age [19,24].

In the central part of the Eastern Anatolia, a higher number of dog bones were identified from the Bronze Age layers of Sos Höyük in Erzurum [25]. However, similar to Arslantepe and the sites in Elazığ region, the Iron Age layers of both Sos Höyük and Büyükardiç in Erzurum showed scarcity of dog bones [18,25]. Paucity of information also appeared from a few dog bones from another Iron Age site, Büyüktepe Höyük, in Bayburt [25,26].

In the eastern most part of the Eastern Anatolia, Iron Age dog remains were mostly found from the Urartian necropolis around the Lake Van region, including a group of dog bones from Altıntepe [17] and a large concentration of dog bones from Yoncatepe Castle [14,23,27].

Despite these multiple reports of dogs at Bronze Age–Iron Age sites in all parts of Eastern Anatolia, knowledge of their biological and cultural status still remains scant. Here, we present a detailed zooarchaeological study of dog bones, including two near-complete dog skeletons, unearthed from the Iron Age residence buildings and workshop complex at Alaybeyi Höyük, Eastern Anatolia. The results of this study reflect both the morphological status and cultural role of Alaybeyi dogs, adding vital information to the existing archaeological knowledge of dogs in Anatolia.

## 2. Materials and Methods

### 2.1. The Site

**Figure 1:**
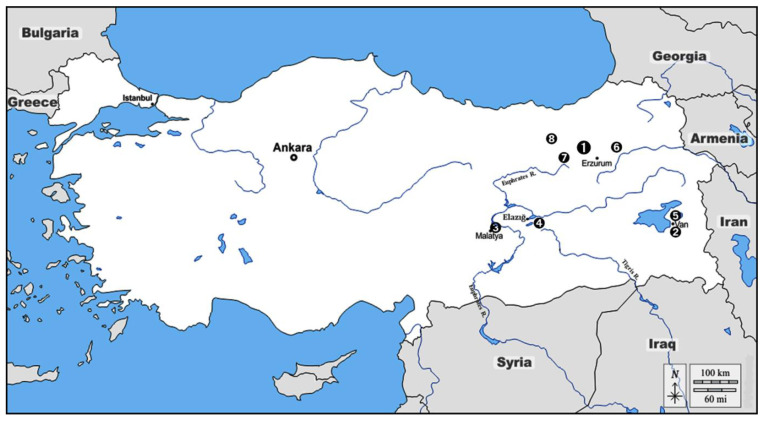
Location of the Eastern Anatolian Bronze Age-Iron Age sites mentioned in the text: 1. Alaybeyi Hoyuk; 2. Yoncatepe; 3. Arslantepe; 4. Iron Age sites under Keban Project; 5. Altıntepe; 6. Sos Höyük; 7. Büyükardiç; and 8. Büyüktepe Höyük (map by A.B. Siddiq).

The archaeological mound Alaybeyi Höyük is located within the border of Alaybeyi village of the Aziziye district, about 28 km northwest of the present Erzurum city, Eastern Anatolia (Figure 1). The site lies on the northwestern part of the large Erzurum plain, between the Kargapazarı Mountains in the north and Palandöken Mountains in the south. At present, the mound has no significant topographical elevation, and therefore was not noticeable to previous archaeological surveys. However, during the construction activities of the Trans-Anatolian Natural Gas Pipeline (TANAP) project, it was suddenly discovered in 2016. The gas pipeline construction was immediately stopped and salvage excavations were conducted by the directorate of the Erzurum Museum in the 2016 and 2017 seasons [28]. To date, only about 3.5–4% of the ~10 hectare site has been excavated along the gas pipeline [29], while the rest of it is still remains unexcavated.

**Figure 2:**
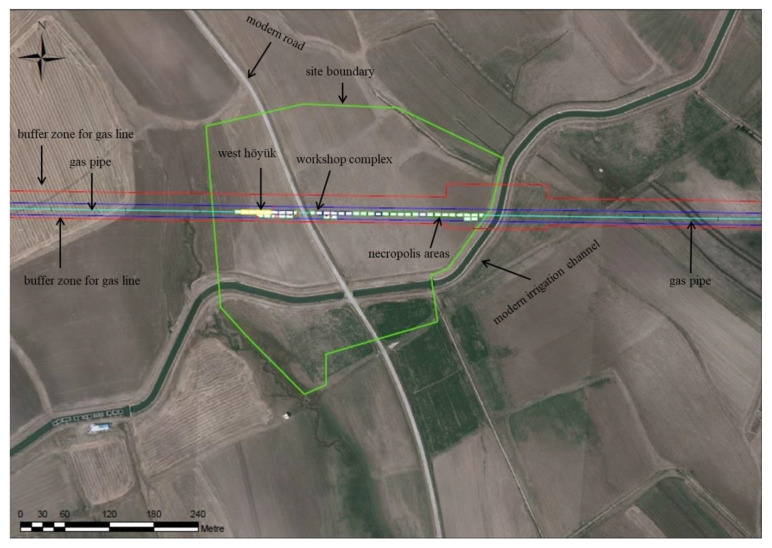
Topography and excavated areas of Alaybeyi Höyük: the modern road divided the site in east and west sections (photo from Alaybeyi Höyük Archive).

The settlement was divided in two sections: east and west (Figure 2). The western section is regarded as the Höyük, which was occupied from Late Chalcolithic period, dated 4721–4553 calibrated BC [29], placing it, so far, as the earliest settlement in northeastern Anatolia. While the Iron Age layers in the west unearthed regular residence architectures, the eastern section revealed a large workshop complex and necropolis, dated from 1000 BC to 200 BC, covering the full period of the Iron Age in Anatolia [30]. Large architectural remains, evidence of extensive production activities, and rich numbers of burials at the large necropolis [29] clearly indicated that Alaybeyi Höyük was a big and important settlement in the region.

### 2.2. Skeletal Remains of the Dogs

**Figure 3:**
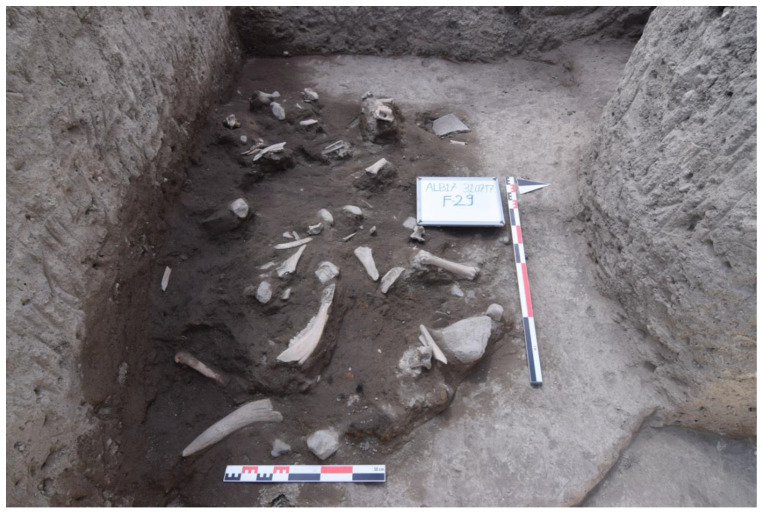
A large composition of animal bones with hearth fragments unearthed at the western section of Alaybeyi Höyük.

A rich assemblage of faunal remains, comprised of a total 4591 identified specimens (NISP), was recorded from Alaybeyi Höyük [20,31]. Most of these bones were hand-collected since it was a rescue excavation with a restricted timeline. Some of the samples were also collected from dry sieving. In many trenches, faunal assemblage was found as a large composition of animal bones (Figure 3), but this did not outnumber the scattered records of faunal remains all over the site, except for the necropolis area. While only a handful of animal bones were found in the Chalcolithic–Bronze Age layers, most of them found from Iron Age layers. Moreover, not a single dog bone was found in the Chalcolithic–Bronze Age layers or in the Iron Age necropolis in the east, leaving the archaeological context for the canid remains associated with only the Iron Age buildings and workshop complex.

With a ratio of 8.87% of NISP, the dog (*Canis lupus familiaris* Linnaeus, 1758) comprised the highest part of the total identified carnivorous species [20,31]. In particular, two dogs were buried at the Early Iron Age workshop complex. Among them, an *in situ* dog skeleton was placed inside the workshop building, which also yielded a pithos (urn) with a child skeleton, an adult human buried in the hocker position, and objects such as large iron slags, pottery remains, bracelets, bowls, and slings [30]. Along with the vertebrae, ribs, carpal and tarsal bones, and phalanges, a total of 172 complete bones were identified from this individual. The other *in situ* dog, buried with its head removed, was found in the courtyard of the workshop, and comprised 54 complete bones, including vertebrae, ribs, and phalanges. The rest of the dog bones were scattered all over the Iron Age building complex and different parts of the workshop. A total of over 300 bone and bone fragments were identified as the remains of the dogs. However, excluding isolated canine teeth, as well as the sternum, carpal and tarsal bones, vertebrae, ribs, phalanges, and claws, a total of 143 specimens—accessible for morphometric analysis—were taken into consideration for this detailed zooarchaeological study.

### 2.3. Estimation of Minimum Number of Individuals (MNI)

Specimens from the two *in situ* dog skeletons were assigned as two individuals followed by an MNI estimation of the remaining specimens, carried out using the methodology proposed by Chaplin (1971) [32].

### 2.4. Morphometry

Z-score calculation was performed to evaluate the size differences of the Alaybeyi dogs and to compare their raw morphometric data with both other Iron Age dogs in Eastern Anatolia and modern dog data. Morphometric data of Yoncatepe (Early Iron Age-Urartian) dogs [14,23,27] were the published archaeological reference of comparison, whereas the average mean of a total of 18 dog breeds—comprising dogs of different typology and size—were used for the comparison with modern dogs (Table 1). In this way, it was possible to determine how many units and in which direction (positive or negative) the Alaybeyi dogs deviated from the average mean of the Yoncatepe Iron Age dogs and the average mean of the modern dog breeds. Z-score values were calculated on the greatest length (GL) values of the bones, considering that GL is accepted to be more effective in predicting the visual morphological traits of a particular species.

### 2.5. Estimation of Height at Withers and Body Weight

Morphometric measurements from long bones, including the humerus, radius, ulna, femur, and tibia, were used for the estimation of visual morphological characters. Multipliers of Harcourt (1974) [33] were used in the estimation of height at withers, and the formulations proposed by Anyonge (1993) [34] were used for the estimation of body weight.

### 2.6. Estimation of Age

The animals’ juvenile or adult statuses were determined by permanent dentition existence as well as on the basis of epiphyseal fusion of the long bones. Among the three skulls examined, the number of alveoli suggested that the dental formula for both dental arches was normal and all permanent teeth had erupted, indicating their status of being the skulls of adult dogs. On the other hand, except for a juvenile individual, epiphyseal fusions in all the long bones were complete, also indicating their status of being the skeletal remains of adult dogs.

### 2.7. Estimation of Sex

Sex estimation was possible for ALB No. 1 (from skull and pelvis), ALB No. 2 (skull), and ALB No. 7 (skull and os penis), while the sex of other individuals remained undetermined due to the absence of a complete skull, pelvis, or os penis (baculum)—the bony structure typical for all carnivores male individuals.

### 2.8. Examination of Pathology

The presence of pathologies and taphonomic modifications on the specimens were examined with the help of Baker and Brothwell 1980 [35].

### 2.9. Examination of Butchery Marks

The presence of all possible butchery modifications, including cut and chop marks, were examined according to methodologies proposed by Chaplin (1971) [32], Davis (1987) [36], and O’Connor (2000) [37]. Detection and observation of any butchery marks were followed in two stages: following the cleaning, first every specimen was examined macroscopically under an illuminated loop; second, in case any marks were suspected, the incision area was closely examined under a light and stereomicroscope, following the methods proposed by Kooi and Fairgrieve (2013) [38] and Domínguez-Rodrigo et al. (2009) [39].

## 3. Results

A total of 143 specimens of dog bones from Alaybeyi Höyük were examined in this study (Table 2). Most of them were in complete or near complete form. In total, 89 of the specimens were identified to a total of 9 distinct individuals. Bones belonging to each of these individuals were grouped under a distinct individual code, while the other specimens were categorized as “ALB miscellaneous” (Table 3). On the basis of the epiphyseal fusions of the long bones, all the identified individuals were found to be adult dogs, except individual ALB No. 6, which appeared to be a young individual.

**Figure 4:**
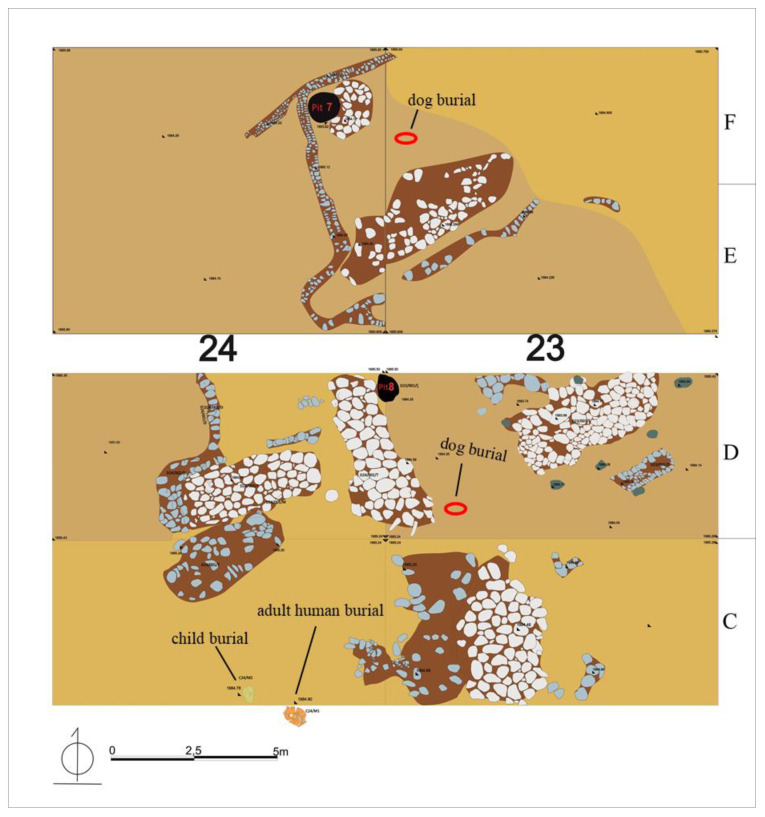
Location of the dog burials from the Iron Age workshop complex: each of Trench D 23 and F 23 respectively revealed single *in situ* dog skeleton (photo from Alaybeyi Höyük Archive).

All dog bones at Alaybeyi Höyük were found from the Iron Age workshop complex in the east section and its courtyard, and the Early–Late Iron Age residence buildings in the west section and their surrounding pits and wells (Table 2). Surprisingly, not a single canine bone was found in the large necropolis. The workshop complex (908–797 cal. BC) was a very large and appears to have been an important building complex, revealed by many small finds, adult human graves, a pithos with a child burial, extensive production materials, and two dog burials (Figure 4).

**Figure 5:**
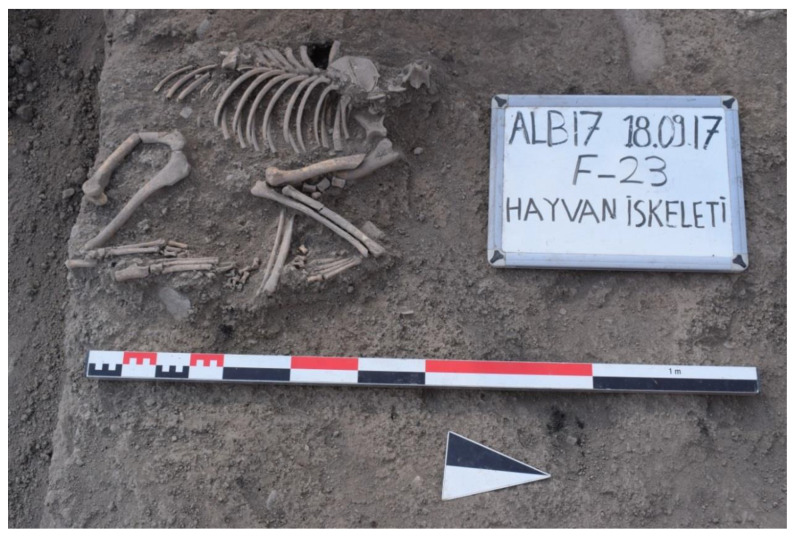
An *in situ* dog burial (ALB No. 1) unearthed from the empty space in the middle of the Iron Age workshop complex at Alaybeyi Höyük.

The central section of the workshop was associated with intensive production activities, supported by smooth stone floor, iron slags, axes, copper alloy tools, a hammer, and bone objects. An *in situ* female dog (ALB No. 1) was unearthed from the empty space in the middle of the workshop, in between the central floor and eastern floor. Most of the skeletal part of the dog, including the sternum, caudal vertebrae, and phalanges, were undisturbed (Figure 5). In the north, the open courtyard of the workshop revealed another *in situ* male dog burial without its skull. No cultural materials were associated with these canid skeletons. However, cultural materials, including ceramic parts, bone objects, and ashes, were found in the north-eastern pit of the courtyard. The open courtyard of the workshop complex was very significant, given that its masonry was the only example at the settlement. A hearth in size of 1 × 1.25 m covered with large ceramic fragments, intact vessels, a bone awl, a stone axe, and mine melting crucible with intense rusty slag residues were also found in the courtyard.

Besides the two dog burials, other canid remains, including skulls, mandibles, humerus, pelvis, sacrum, and tibia, were found scattered in the workshop and courtyard complex (Table 2). Among them, one skull appeared to be the skull of the dog buried without its head.

The “garbage pits”—a characteristic feature of Anatolian Iron Age settlements—of Alaybeyi Höyük also yielded a considerable number of dog bones. Particularly, three Early Iron Age pits and one Early Iron Age well, associated with the habitation architectural remains in the western section, unearthed a considerable number of dog bones, which are associated with other animal bones and cultural artifacts. Among them, one pit revealed two dog mandibles, a dog ulna, and a large number of young goat bones. Dog bones from the Late Iron Age period were also mostly concentrated with other animal bones, found scattered in the residence buildings. Skulls, isolated canine teeth, a humerus, radius, ulna, and tibia were the most notable specimens (Table 2).

Height at withers and body weight of the Alaybeyi dogs were estimated from long bones such as humerus, radius, ulna, femur, and tibia. Due to the missing skeletal parts, estimation of mentioned parameters was possible to perform for seven individuals. The calculated data present an average height at withers of the Alaybeyi dogs to be 60.49 cm—with the lowest height at withers being 57.46 cm for ALB No. 2 and the highest height at withers being 64.23 cm for ALB No. 7 (Table 4).

The average body weight of the Alaybeyi dogs observed was 34.54 kg—with the lightest body mass 21.54 kg for the comparatively young dog ALB No. 6 and the heaviest body weight 45.44 kg for the adult individual ALB No. 7 (Table 4). In general, five dogs at Alaybeyi Höyük were observed to be “large-sized” dogs. However, considering the estimation of the visual morphological characters from the height at withers and body weight, ALB No. 7 was found to be a much larger individual than the others—possibly due to being both a male and a breed close to the mastiff type.

Z-score calculations were made to evaluate the size differences between the Alaybeyi dogs and other Iron Age dogs in the region, as well as the average mean of modern dog breeds. The Z-score of Alaybeyi dogs were separately compared with the Z-score of the Yoncatepe Early Iron Age–Urartian dog breeds (Table 5) and the score of the modern dog breeds (Table 6). The GL values were used in the evaluation of the Z-score calculations since GL is more commonly preferred in predicting height at withers among other visual morphological characters. It was found that, except for one individual, ALB No. 2, there was a positive deviation in the GL values of the Alaybeyi dogs from the general average GL value of the Yoncatepe dogs. ALB No. 2 was found to be deviating from the mean by −0.600 units in the negative direction. ALB No. 7 dog was the individual with the highest deviation, also probably due to its larger size and being a male individual.

The comparison between the Alaybeyi dogs and the average mean of the modern dog breeds also revealed a similar picture. However, the positive deviation of ALB No. 7 from the mean value of the modern dog breeds was not as much as found in the comparison with the Yoncatepe dogs, probably due to the presence of large dog breeds, such as the Great Dane, Mastiff, and St. Bernard, among the modern dogs group.

**Figure 6:**
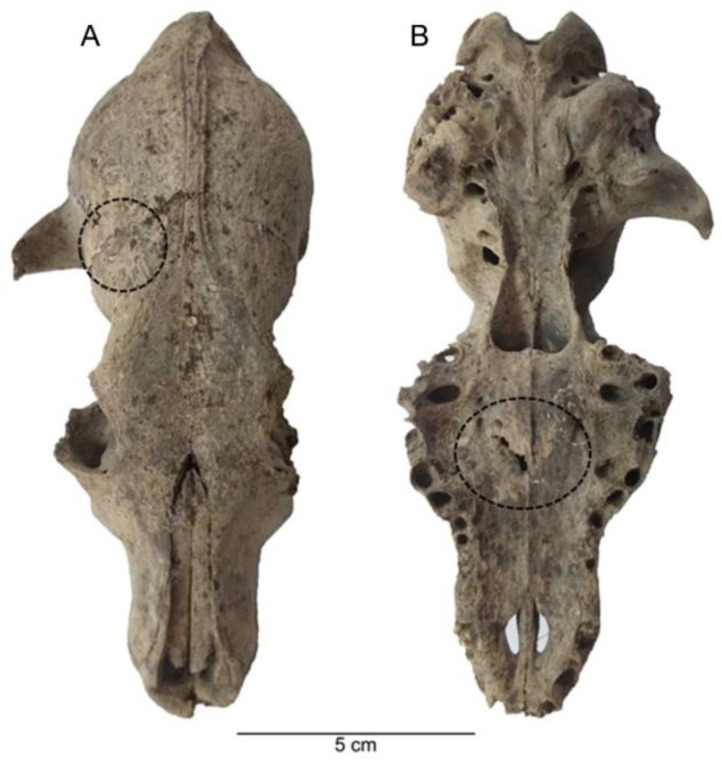
A dog skull from Alaybeyi Höyük showing frontal and palatal healing fractures: (**A**) frontal; (**B**) palatal.

**Figure 7:**
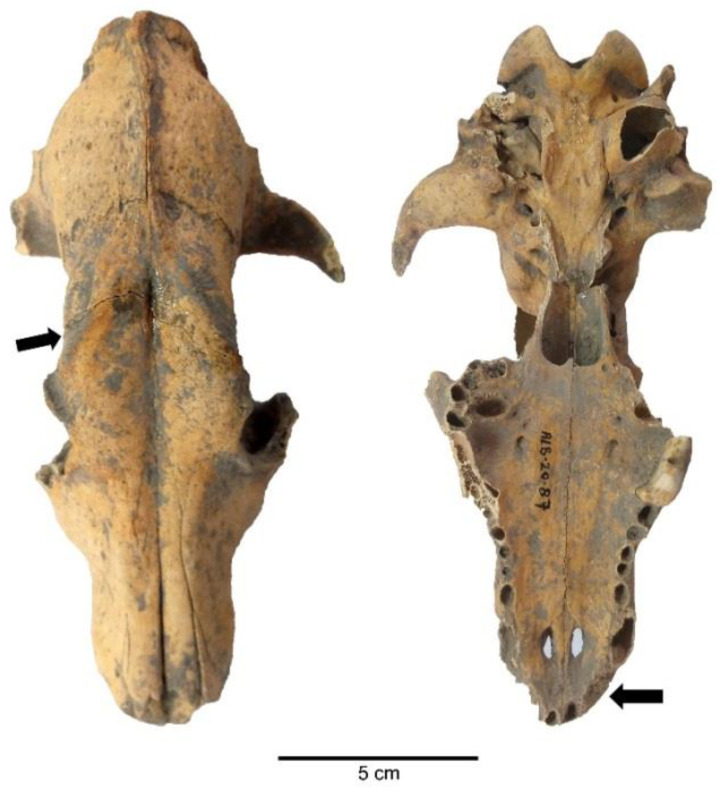
A dog skull from Alaybeyi Höyük with a healing fracture (**left arrow**) and oligodontia (**right arrow**).

**Figure 8:**
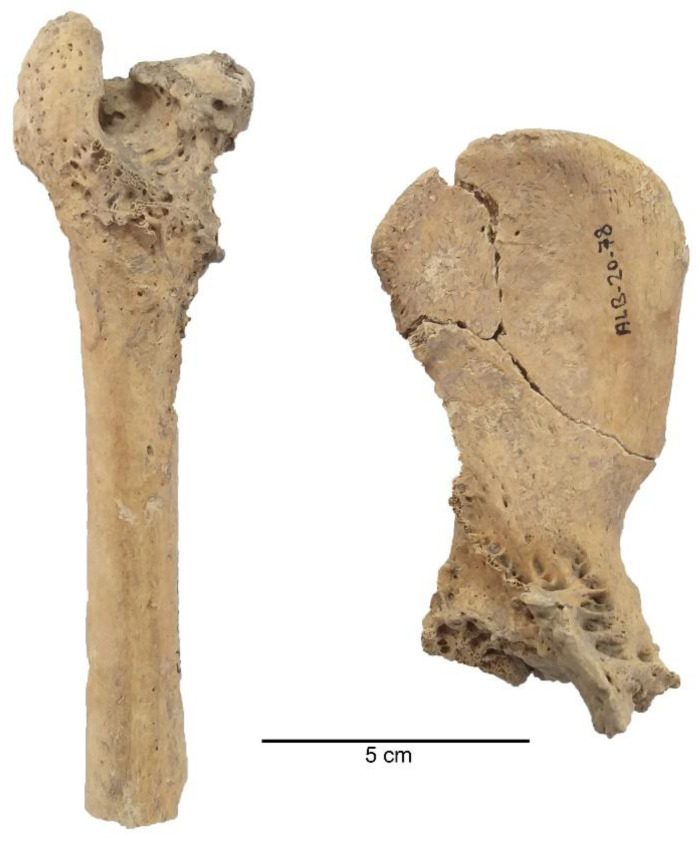
Example of coxal dysplasia and osteophyte proliferations in the Alaybeyi dog bones—these two specimens apparently belonged to a single individual: (**left**) femur; (**right**) os coxae.

Pathological marks were observed in 4.90% (NISP = 7) of the sample studied. Pathologies such as alveolar recession in the teeth and oral region, a healing fracture in the palatum durum (hard palate), a healing fracture in the frontal bone, oligodontia, periodontal disease, coxal dysplasia, osteophyte proliferations, and exostoses were the most notable (Figure 6, Figure 7 and Figure 8).

**Figure 9:**
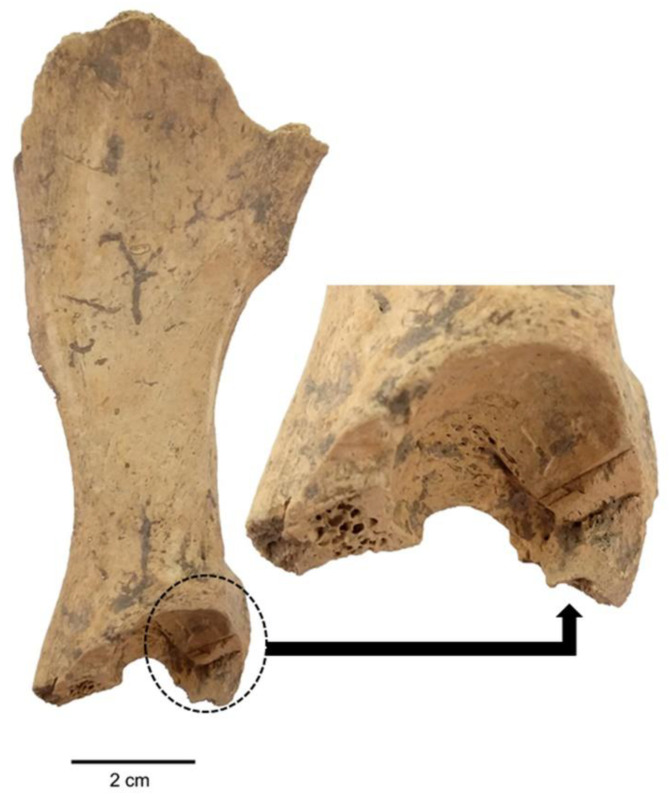
Cut and chop marks in the right coxal bone of an Alaybeyi dog.

**Figure 10:**
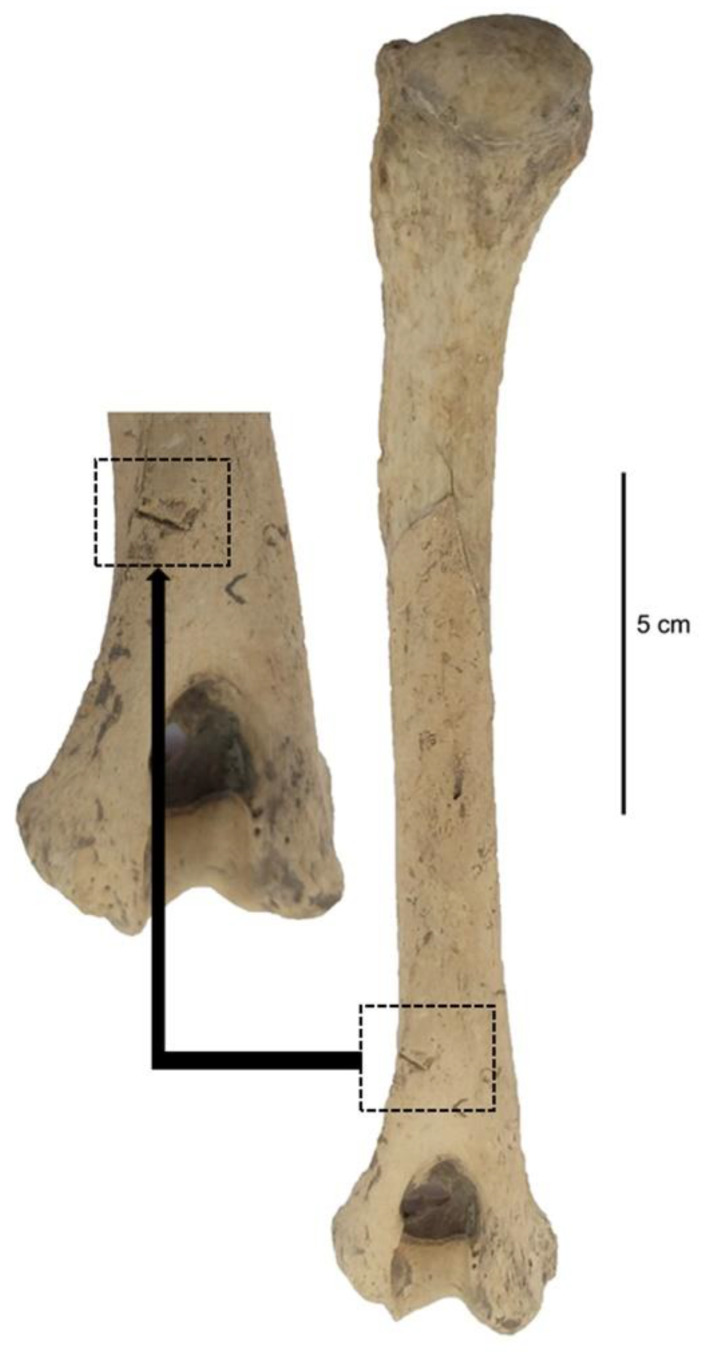
The left humerus of the dog “ALB No. 4” from Alaybeyi Höyük exhibits a deep cut mark.

Two of the specimens, a coxae and a humerus, were observed with cut and chop marks. The incisions appeared to be V-shaped cut lines when examined under a stereo microscope. Although these two specimens were unearthed from two different trenches, it was possible that they belonged to the same individual given their close location. However, this is yet to be confirmed. Both cut and chop marks were observed in the coxae (Figure 9), unearthed from Trench D-23 and was evaluated under the “ALB miscellaneous” group. The os coxae fragment was cut obliquely from the acetabulum part with a large knife or chopper. Considering the visual dimensions, it appeared that the coxae fragment belonged to a large dog. On the other hand, a deep cut mark was observed in the distal part of the humerus (Figure 10), which belonged to the dog examined as ALB No. 4, unearthed form Trench E-23. Apparently, to be a cut caused by a sharp knife, the mark was observed towards the epicondylus in the distolateral part of the humerus. With the help of the measurements of the humerus, the dog (ALB No. 4) was estimated to be 59.46 cm in height at withers and 29.67 kg in body weight. Notably, both specimens with cut marks were unearthed from the Early Iron Age workshop complex (900 BC—800 BC), where the largest concentration of dog bones occurred along with the presence of two *in situ* dog burials.

## 4. Discussion

Since the Neolithic, dogs have been valued by pastoral and agricultural groups in Anatolia for their usefulness in shepherding and guarding [10,11,40]. In many Anatolian sites, dogs were often buried in an intra-settlement context [5,12,16], associated with humans and other animals [9,14,41], or sometimes buried in the same alignment as human burials [15]. In others cases, dogs bones were unceremoniously discarded in general waste deposits [18,19,20]. Dogs were also associated with fertility deities, healing, the underworld, and the afterlife—evident from multiple cases of dog burials and ritual sacrificing of dogs in the Bronze Age and Iron Age sites [5,13,16,21]. This indicates that dogs played a multifunctional role in Anatolia throughout ancient times [40].

In Eastern Anatolia, dogs appeared to be more common from the Chalcolithic and Bronze Age periods—perhaps associated with extensive animal husbandry. Particularly, there was a dramatic increase in domestic dog in the Bronze Age, comprising up to 10% of the total identified fauna at some sites [12,22]. Nevertheless, a sharp decrease in the ratio of dogs was observed at most of the Iron Age sites in the east and central parts of East Anatolia, comprising only less than 2% of the total identified fauna [19,24,26]. In the Erzurum region, some Iron Age sites present only a small number of dog bones. For example, only a few dog bones, including a mandible and long bones of a 1–5-month-old puppy, were found from the Early Iron Age site Büyükardiç [18]. The Iron Age levels of Sos Höyük in Erzurum also present only very few dog bones [25,26]. Scarcity of canine remains also appeared at the Iron Age site Büyüktepe Höyük in Bayburt [26]. In contrast, domestic dogs still stand high at Alaybeyi Höyük, comprising about 9% of the total identified fauna [20].

Dog remains also comprised significant ratio at the Iron Age sites in the eastern parts of Eastern Anatolia, mostly associated with Urartian castles and royal settlements in the Van region [14,17,42]. However, the Iron Age dogs in the Malatya region were associated with the settlements under Neo-Hittite influence [12], and the Iron Age dogs from the Elazığ region were associated with a series of simple village type settlements established by the people moving away from the central authority following the collapse of the Hittite Empire [24,30,43]. Archaeological dogs in the Lake Van region were often concentrated with necropolises. For instance, a group of dog bones, including remains of a puppy, were identified from the Urartu–Iron Age necropolis of Altıntepe [17]. The largest concentration of dog remains, including complete dog skeletons associated with human burials, comes from the Early Iron Age royal necropolises of the Van-Yoncatepe Castle [14,23]. In contrast, almost all the dog bones in the Erzurum, Elazığ, and Malatya region were associated with household buildings and activity areas [19,20,24,26]. Here, although showing strong contrast regarding the ratio of the dogs in the total identified fauna, Alaybeyi too showed no exception to the Iron Age sites in the central and western part of Eastern Anatolia—given that not a single dog bone was unearthed in the necropolis area [20].

Although sporadic, dog bones were commonly present almost every Iron Age site in Eastern Anatolia. Except for the Early Iron Age Van-Yoncatepe [14], canine remains from all of these sites were only reported with their numbers and without any particular attribution on their morphological, biological, and cultural status. Therefore, Van-Yoncatepe dogs were the only archaeological benchmark in the region to compare to the Alaybeyi dogs. While the height at withers of the Van-Yoncatepe dogs was calculated between 52.15 cm and 60.13 cm [27], the calculated data presents the height at withers for Alaybeyi dogs between 57.46 cm and 64.23 cm. On the other hand, the body weight of the Van-Yoncatepe dogs was calculated between 19.99 kg and 29.64 kg [23], whereas the calculated data presents the body weight of Alaybeyi dogs between 21.54 kg and 45.44 kg. Hence, though the Van-Yoncatepe dogs were categorized in the group of large-size races [23,27], with an average height at withers of 60.49 cm and an average body weight 34.54 kg, the Alaybeyi dogs stand to be larger than those in Van-Yoncatepe. Particularly, though the lightest body mass 21.54 kg comes from a young individual, at least five dogs at Alaybeyi Höyük were estimated to be much heavier and “larger-sized” dogs, with a very large individual (ALB No. 7) showing a height at withers of 64.23 cm and a body weight 45.44 kg. Considering the estimation of the visual morphological characters from the height at withers and body weight, the very large feature of ALB No. 7 was possibly because of being both a male and a breed close to the mastiff type [44].

The comparison between the Alaybeyi dogs and the average mean of the modern dog breeds also revealed a similar situation. However, the positive deviation of ALB No. 7 from the mean value of the modern dog breeds was not as much as found in the comparison with Yoncatepe dogs, probably due to the presence of large dog breeds, such as the Great Dane, Mastiff, and St. Bernard, among the modern dogs group.

Ethnographically, it is clear that there is a wide range of human–dog relations [45]. Together with their roles as companions, hunting assistants, or guardians of the herds [46], dogs are often raised for food, sources of meat in crisis times, specific rituals, or medicinal purposes [46,47,48]. Puppy sacrifice was in practice in Anatolia during the Bronze Age, and both sacrifice of the dogs and cynophagy is known from Iron Age Eastern Mediterranean [21,40,49,50,51]. Particularly, dog remains found in cooking pots from the Iron Age stratum of the Sardis on the Aegean coast of Western Anatolia were interpreted as sacrificial meals [51]. Dog bones with cut marks from the Early–Middle Iron Age stratum of Tel Miqne-Ekron in Palestine [49] and Ashkelon in Israel [50] were also described as evidence of cynophagy, sacrificial use, and the sacred status of dogs. At Alaybeyi Höyük, the distribution of dog bones was often associated with ungulate bones deposited as food residues, indicating that dog bones too might have a similar function. Although cynophagy was not noticed at other neighboring Iron Age sites, it appears that the practice of eating dog meat was present at Alaybeyi Höyük, if not a regular diet, at least for a special occasion. Cut and chop marks on the specimens from the important workshop building and its courtyard appeared to be best support for this. The “garbage pits” of Alaybeyi Höyük—a characteristic of Iron Age settlements in Anatolia—also yielded a considerable number of dog bones often associated with ungulate bones [20], further raising aspiration to think that the dog bones were part of food residues. On the other hand, so far no archaeological and zooarchaeological evidence suggested that dogs were sacrificed at the site. Hence, occasional cynophagy appeared to the option; however, other possibilities, including ritual uses or execution of bone tools, could be of importance in future studies with a more elaborate sample size.

In the evaluation of pathological marks, one specimen was observed with coxal dysplasia, two skulls were observed with healing fractures in the palatum durum (hard palate) and frontal region as well as alveolar recession and oligodontia in the oral region, one specimen was observed with periodontal disease, and a long bone was observed with a bone tumor [35]. These pathologies were formed during the life-time of the dogs. Pathologies in the oral cavity and dentition indicate that these animals were probably malnourished and had to live on solid food, such as bones. On the other hand, humans at Alaybeyi Höyük appear to be responsible for the healing fractures in the frontal region—given that humans might have hit the dogs to control them or prevent their aggressive behavior [35].

Dogs might have also played an important role in handling herds and guarding at Alaybeyi Höyük. The ratio and pray choice in the wild game [20,31] do not indicate that the inhabitants at the site often required dogs for hunting activities. Hence, guarding and shepherding would be the best fit to their roles. This imposed on the Iron Age people at Alaybeyi Höyük who were mostly engaged with extensive production activities [28]. It appears that the Alaybeyi dogs were generally large-sized and of the Mastiff type [44]; indeed, this breed is still preferred by the shepherds and cattle herders in Eastern Anatolia.

On the other hand, the two dog burials in the workshop complex—one of the most important places at the site—may indicate the special importance of dogs at Alaybeyi Höyük. Installation of near-complete dog skeletons in special deposits, such as the workshop complex, and installation of dog bones mostly in habitation architectures and their associated deposits, could be the best evidence for this. Particularly, the *in situ* status of these dog skeletons stand as strong indication that, unlike practices of throwing away the dead shepherd dogs in present southeast and east Anatolia [31,46], the two dogs at the workshop complex were intentionally buried by the people at the site. However, there is no indication that the Alaybeyi dogs were regarded as pets—given that none of them were buried with humans or in the manner of any human burials, nor associated with any kind of special artifacts.

## 5. Conclusions

In conclusion, morphologically the Alaybeyi dogs stand to be larger than the Van-Yoncatepe dogs—apparently taking position in the Mastiff dog group. It is evident that dogs were not raised at Alaybeyi Höyük as food; yet, unlike the other Iron Age sites in the region, dogs were apparently consumed at least for occasional purpose. Considerable pathological marks indicate that some of the dogs were undernourished and injured by humans. Nevertheless, the two *in situ* dog skeletons at the Iron Age workshop complex indicate the special importance of dogs. Despite only 3–4% of the site having been excavated under the two years of salvage excavations, Alaybeyi Höyük presented far richer canine remains to its neighboring Iron Ages sites in Eastern Anatolia, adding vital information to the very limited archaeological knowledge of dogs in Anatolia.

## Figures and Tables

**Table 1:** List of the modern dog breeds used in comparison with Alaybeyi dogs.

Dog Breeds	Number	Dog Breeds	Number
French bulldog	1	St. Bernard	2
Pekingese	1	Rotweiller	2
Boxer	3	Kangal dog	3
Terrier	3	Irish setter	1
Cocker spaniel	1	German shepherd	5
Clumber spaniel	1	Pointer	1
Canaan dog	1	Siberian husky	1
Great Dane	1	Doberman	3
Mastiff	1	Mixbreed	6

**Table 2:** Distribution of the anatomical elements of the Alaybeyi dogs examined in this study.

	Early-Middle Iron Age													Late Iron Age					
Archaeological Context	Workshop Complex					Pit 2	Pit 1	Well 1	Structure B		Pit 4	Structure A	Courtyard	Structure A			Courtyard	Architectural Remains	
Trench	D 23	E 23	F 23	E 24	F 24	E 27	F 27	E 32	F 28	E-F 29	F 29	E 30	E 31	E 28	E 29	F 30	F 31	F 32	NISP
Skull	1	1	1				1				1		1		1				7
Mandible	2	1	2			2			1	1	1								10
Atlas	1	1	1														1		4
Axis	1		1					2	1			2							7
Sacrum		2	1																3
Scapula	2		2	1															5
Humerus	2	4	2	1	1						1			1	1				13
Radius	2		2				2					1				1	1		9
Ulna	2		2			1													5
Metacarpus	7	1	9		1				1			1							20
Pelvis	1	2	1	1	2														7
Baculum			1																1
Femur	2	1	2																5
Patella	3		2																5
Tibia	3	2	4	1			1		1						2			2	16
Fibula	2		2																4
Calcaneus	1		2																3
Talus	1		2																3
Metatarsus	5		8	1			1					1							16
**Total**	38	15	47	5	4	3	5	2	4	1	3	5	1	1	4	1	2	2	143

**Table 3:** Distribution of individuals identified from the assemblage.

Individual No.	Number of Bones
ALB No. 1	45
ALB No. 2	1
ALB No. 3	1
ALB No. 4	1
ALB No. 5	1
ALB No. 6	1
ALB No. 7	37
ALB No. 8	1
ALB No. 9	1
ALB miscellaneous	54
NISP	143

**Table 4:** Distribution of the sex, height at withers, and body weight of the Alaybeyi dogs.

Individual No.	Sex	Height at Withers	Body Weight
ALB No. 1	Female	60.90	39.19
		H, R, Fe, Ti	H, Fe
ALB No. 2	Male	57.46	27.77
		H, R, Fe, Ti	H, Fe
ALB No. 3	Undetermined		43.64
			Fe
ALB No. 4	Undetermined	59.46	29.67
		H	H
ALB No. 5	Undetermined	60.43	
		R	
ALB No. 6 *	Undetermined		21.54
			H
ALB No. 7	Male	64.23	45.44
		H, R, U, Fe, Ti	H, Fe

* Young individual; H—humerus; R—radius; U—ulna; Fe—femur; Ti—tibia.

**Table 5:** Osteometric measurements of the Alaybeyi dogs and their comparison with the Yoncatepe dogs.

	Skull				Humerus		Radius			Ulna			Femur			Tibia		
	N	TL	Z Score	N	GL	Z Score	N	GL	Z Score	N	GL	Z Score	N	GL	Z Score	N	GL	Z Score
ALB No. 1				2	184.76 *	0.481	2	183.65 *	0.246				2	201.24 *	0.675	2	204.42 *	0.521
ALB No. 2				1	175.25	−0.600												
ALB No. 4				1	181.09	0.064												
ALB No. 5							1	183.91	0.269									
ALB No. 7				1	195.04	1.648	2	192.65 *	1.032	2	226.71 *	1.468	1	214.12	1.778	2	219.32 *	1.680
ALB No. 8	1	165.87	−2.637															
ALB No. 9	1	198.61	0.089															
YT M6 **	18	199.24		35	180.52		49	180.72		37	205.81		37	192.9		45	197.48	
YT M5 ***	1	206.55		1	166.86		1	168.43		1	185.35		1	181.61		1	180.23	

*: Average of the right and left bones; **: Individual dog skeleton unearthed from grave M6 at Yoncatepe castle ([14], and unpublished data); ***: Individual dog skeleton unearthed from grave M5 at Yoncatepe castle ([14], and unpublished data).

**Table 6:** Osteometric measurements of the Alaybeyi dogs and their comparison with average mean of modern dog breeds.

	Skull				Humerus		Radius			Ulna			Femur			Tibia		
	N	TL	Z Score	N	GL	Z Score	N	GL	Z Score	N	GL	Z Score	N	GL	Z Score	N	GL	Z Score
ALB No. 1				2	184.76 *	0.217	2	183.65 *	0.113				2	201.24 *	0.204	2	204.42 *	0.182
ALB No. 2				1	175.25	−0.051												
ALB No. 4				1	181.09	0.114												
ALB No. 5							1	183.91	0.119									
ALB No. 7				1	195.04	0.507	2	192.65 *	0.334	2	226.71 *	0.364	1	214.12	0.517	2	219.32 *	0.525
ALB No. 8	1	165.87	−1.113															
ALB No. 9	1	198.61	−0.141															
Modern dog **	37	204.49			176.29			178.45			208.92			192.04			195.67	

*: Average of the right and left bones; **: Consisted of the average mean of 18 different modern breeds.

## Data Availability

The dog bones from Alaybeyi Höyük are available in the collections of the Zooarchaeology Laboratory, Department of Anthropology, Mardin Artuklu University, Turkey, and available for further study via application through the General Directorate of Cultural Heritage and Museums, Ministry of Culture and Tourism, Republic of Turkey. The datasets generated, analyzed, and used in this study will be provided by the corresponding author on request.
